# Effects of K11R and G31P Mutations on the Structure and Biological Activities of CXCL8: Solution Structure of Human CXCL8_(3-72)_K11R/G31P

**DOI:** 10.3390/molecules22071229

**Published:** 2017-07-21

**Authors:** Hsi-Tsung Cheng, Hui-Yuan Yu, John R. Gordon, Fang Li, Jya-Wei Cheng

**Affiliations:** 1 Institute of Biotechnology and Department of Medical Science, National Tsing Hua University, Hsinchu 300, Taiwan; habercheng@gmail.com (H.-T.C.); gwalt1103@gmail.com (H.Y.-Y.); 2 Division of Respirology, Critical Care and Sleep Medicine, Department of Medicine, University of Saskatchewan, Saskatoon, SK S7N 5B4, Canada; john.gordon@usask.ca; 3 Department of Immunology, Dalian Medical University, Dalian 116044, China

**Keywords:** CXCL8, antagonist, NMR, structure, mutation

## Abstract

The ELR-CXC chemokines are important to neutrophil inflammation in many acute and chronic diseases. Among them, CXCL8 (interleukin-8, IL-8), the expression levels of which are elevated in many inflammatory diseases, binds to both the CXCR1 and CXCR2 receptors with high affinity. Recently, an analogue of human CXCL8, CXCL8_(3–72)_K11R/G31P (hG31P) has been developed. It has been demonstrated that hG31P is a high affinity antagonist for both the CXCR1 and CXCR2. Herein, we have determined the solution structure and the CXCR1 N-terminal peptide binding sites of hG31P by NMR spectroscopy. We have found that the displacement within the tertiary structure of the 30 s loop and the N-terminal region and more specifically change of the loop conformation (especially H33), of hG31P may affect its binding to the CXCR1 receptor and thereby inhibit human neutrophil chemotactic responses induced by ELR-CXC chemokines. Our results provide a structural basis for future clinical investigations of this CXCR1/CXCR2 receptor antagonist and for the further development of CXCL8 based antagonists.

## 1. Introduction

CXCL8 (Interleukin-8, IL-8) and other ELR-CXC chemokines (CXCL5 [ENA-78], CXCL6 [GCP-2], CXCL7 [NAP-2], CXCL1 [GROα], CXCL2 [GROβ], and CXCL3 [GROγ]) are small proteins (70–80 amino acids) that play important roles in neutrophil chemotaxis, activation and exocytosis in inflammatory diseases [1]. This subfamily of chemokines all possesses a tri-peptide motif of ELR at the NH_2_ terminus, proximal to a CXC motif. CXCR1 and CXCR2 are the specific receptors that have been found for ELR-CXC chemokines [2]. CXCL8 binds to CXCR1 and CXCR2 with high affinity, whereas other members bind with lower affinities to either CXCR1 or CXCR2. Clinically, elevated plasma levels of CXCL8 and other ELR-CXC chemokines have been found with chronic diseases such as arthritis, chronic obstructive pulmonary disease (COPD), asthma, cystic fibrosis, atherosclerosis, inflammatory bowel disease (IBD), psoriasis and cancer [3] as well as in acute indications such as reperfusion injury and acute respiratory distress syndrome (ARDS) [1].

The three dimensional structures of CXCL8 have been determined by NMR spectroscopy [4] and X-ray crystallography [5]. CXCL8 forms a homologous dimer with each monomer consisting of three anti-parallel β-strand connected with loops and one *α*-helix at C-terminal which lays back on the β-sheet. The four cysteines form two disulphide bonds (Cys^7^ to Cys^34^ and Cys^9^ to Cys^50^) and keep two amino-terminal regions together [4]. These two disulphide bonds are confirmed to be essential for receptor recognition and biological activity. Previous studies have indicated that residues E4-R6, I10-I22, and S30-A35 of CXCL8 are essential for receptor binding and activation and that the C-terminal α-helix is important for stabilizing the tertiary structure [6,7,8,9,10]. 

Li et al. have reported that bovine CXCL8_(3–73)_K11R/G31P (bG31P), an analogue of CXCL8 with the mutations of CXCL8 at K11 and G31 positions, is a high affinity antagonist against CXCR1 and CXCR2 [11,12,13]. It has also been shown that human G31P (hG31P) effectively blocks the abilities of ELR-CXC chemokines to activate or chemoattract neutrophils *in vitro*, and further demonstrated that it is an effective antagonist in vivo [14,15]. Moreover, hG31P has been demonstrated to restrict lung cancer growth by inhibiting tumor cell proliferation, metastasis, the acquisition of resistance to chemotherapeutic agents, and suppressing angiogenesis [16]. However, no detailed structural information of this double-mutant has been provided. In here, we have determined the effects of K11R and G31P mutations on the structure and biological activities of CXCL8. Our results provide a structural basis for future development of this CXCR1/CXCR2 receptor antagonist.

## 2. Results and Discussion

### 2.1. NMR Spectroscopy

**Figure 1:**
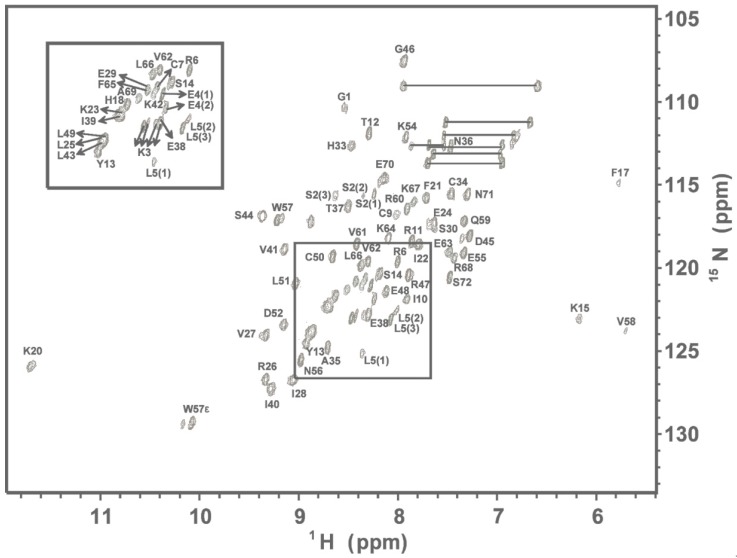
^1^H-^15^N HSQC of hG31P. Cross-peaks are labeled according to the residue types and numbers.

**Figure 2:**
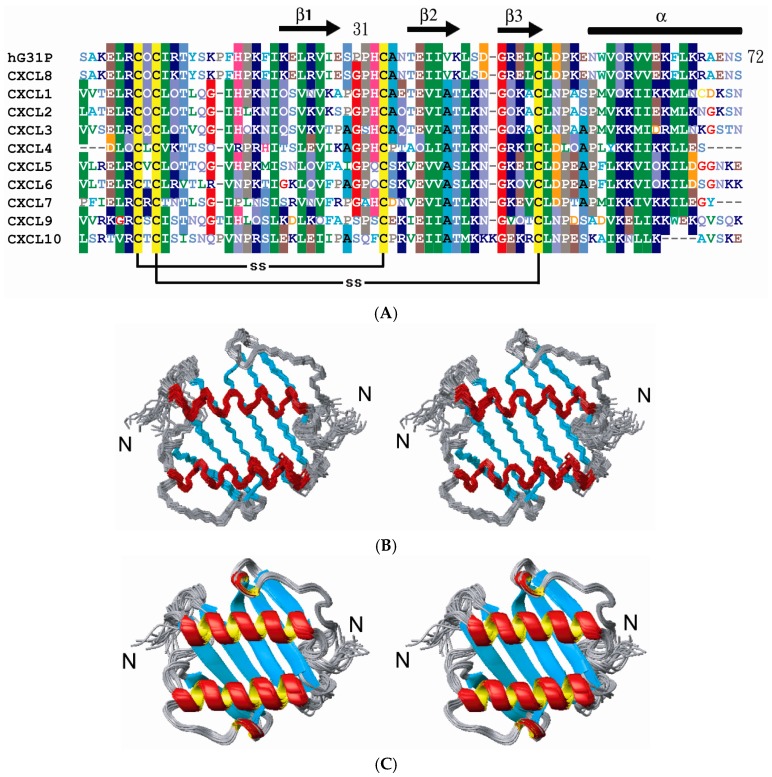
(**A**) A multiple sequence alignment of hG31P and CXCL1 to CXCL10. Secondary structural elements are shown above the alignment; (**B**) A stereoview of the backbone superimposition of the final 20 structures; (**C**) Ribbon diagram of the final 20 structures of hG31P.

The % assignment of backbone ^1^H, ^15^N, ^13^C, and ^13^CO resonances for the 72 residue hG31P is 98% and for side chain resonances is 96% (Figure 1). A total of 2343 NOE-derived distance constraints, including 532 intraresidue, 669 sequential, 352 medium (1 < i-j < 5), 465 long-range (i-j ≥ 4), 99 intermolecular, and 120 hydrogen bond distance restraints in conjunction with 106 backbone dihedral angles are used in the structure calculations. Multiple sequence alignments with secondary structural elements for hG31P and CXCL1 to CXCL10 are shown in Figure 2A. The overlay of the backbone atoms for the 20 lowest energy structures and the ribbon diagram of the lowest energy structure of hG31P are shown in Figure 2B,C. The energetic and structural statistics are listed in Table 1. The rmsd calculated from the averaged structured region for hG31P is 0.68 Å for the backbone heavy atoms (N, C, and C_α_) and 1.38 Å for all heavy atoms.

### 2.2. Structure Comparison with CXCL8

**Figure 3:**
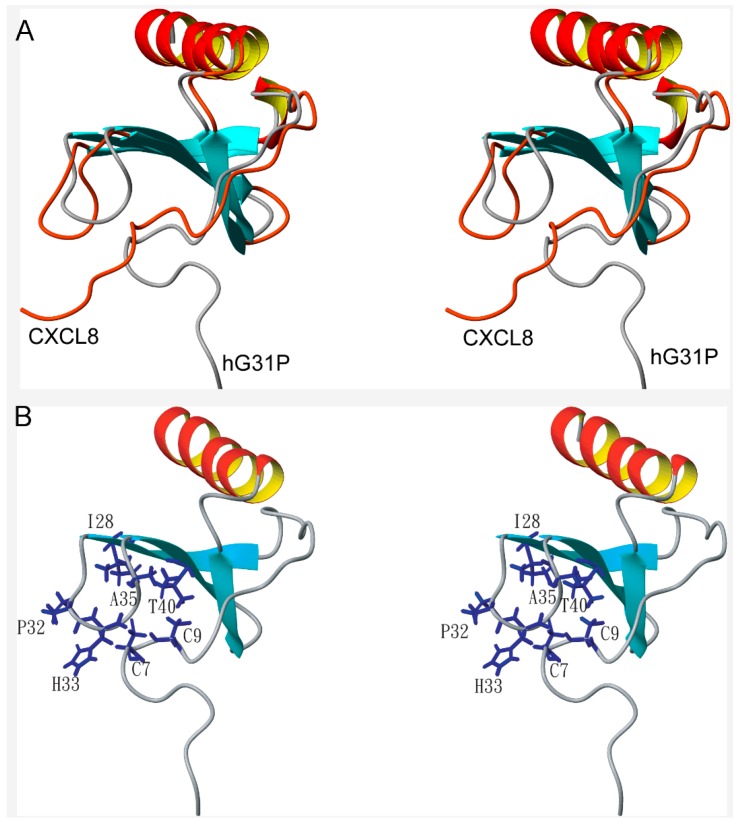
(**A**) Stereo view of the superposition of the ribbon structures of hG31P (cyan) and CXCL8 (PDB code 1ILP; brown) showing the displacement of the ELR N-terminal region and the E29–T37 loop between hG31P and CXCL8. The structures were superimposed by residues F21–E29, G46–L51, and N56–S72; (**B**) Stereo view of the schematic representations of the residues involved in inter-residue NOEs of hG31P. None of such NOEs were found in CXCL8.

**Figure 4:**
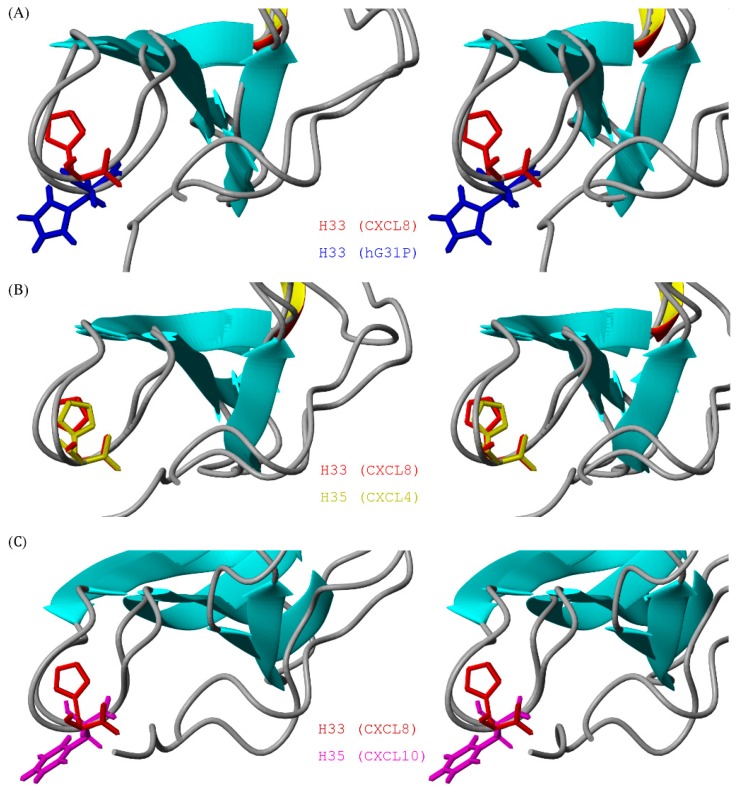
Superposition of the ribbon structures of the 30 s loop between (**A**) CXCL8 (PDB: 1IL8, H33 in red) and hG31P (H33 in blue); (**B**) CXCL8 (PDB: 1IL8, H33 in red) and CXCL4 (PDB: 1F9Q, H35 in green); (**C**) CXCL8 (PDB: 1IL8, H33 in red) and CXCL10 (PDB: 1LV9, H35 in pink).

The solution structure of hG31P forms a dimer with each monomer consisting of three antiparallel β-strands (residues I22–E29, T37–L43, R47–D52) and one α-helix (residues N56–S72) which is similar to the structure of CXCL8 (Figure 2). However, there are localized differences between these two proteins [6,7,8,9,10]. It has been shown that residues E4–R6, I10–I22, and S30–A35 of CXCL8 are essential for receptor binding and activation [17]. Comparing the structures of hG31P and CXCL8 reveals that the E29–T37 loop and the N-terminal region of hG31P are more close to the β-strands than the regions of CXCL8 (Figure 3A). In hG31P, NOEs were observed between residues C7/H33 and Q8/I28 (Figure 3B). On the other hand, NOEs between residues Q8/I40 were found in CXCL8. There are long range NOEs between residues S14–F17 and the side chain of W57 of hG31P. None of such NOEs were found in CXCL8. In addition, the histidine residue at position 33 was forced to point out from the loop of hG31P comparing with the same residue in CXCL8 (Figure 4A). This difference was due to the fact that the mutated proline residue at position 31 occupied the space of H33 in the loop. The mutations of K11R and G31P of hG31P apparently cause the displacement of the E29–T37 loop and the N-terminal region and change of the loop conformation (especially H33), hence affecting its binding to the CXCR1 receptor and inhibit human neutrophil chemotactic responses induced by ELR-CXC chemokines.

### 2.3. Peptide Binding

**Figure 5:**
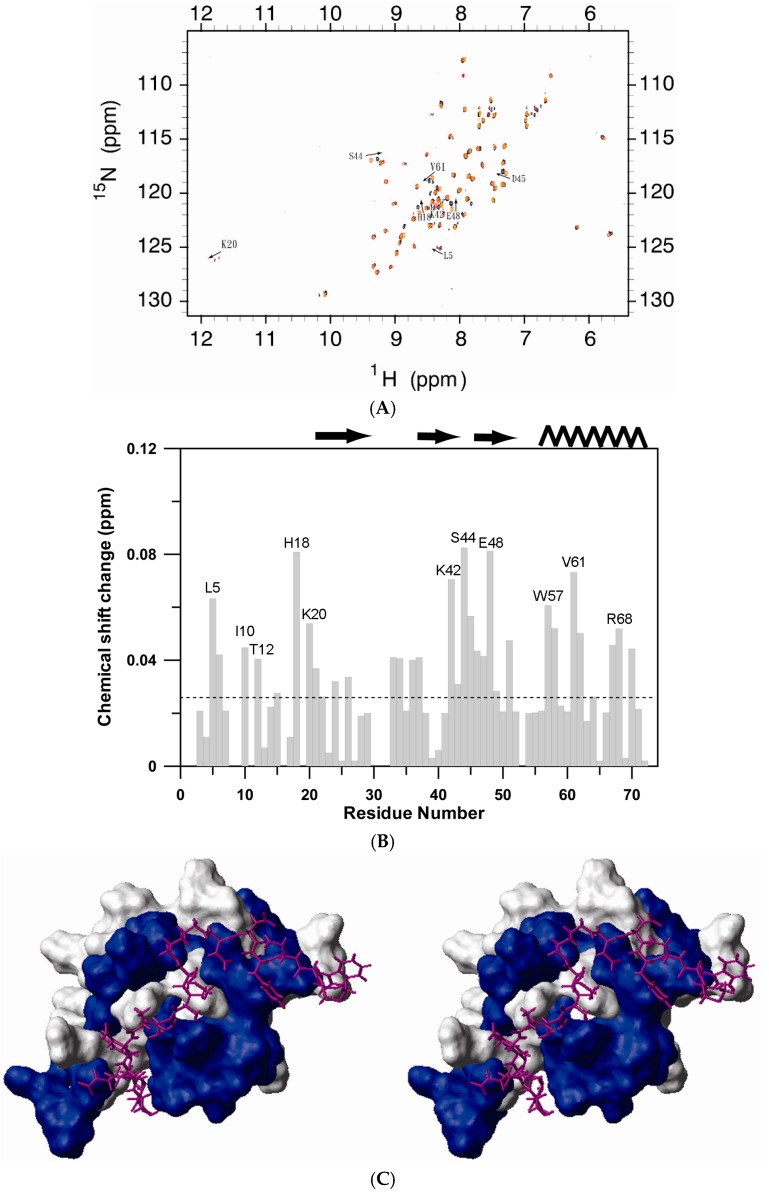

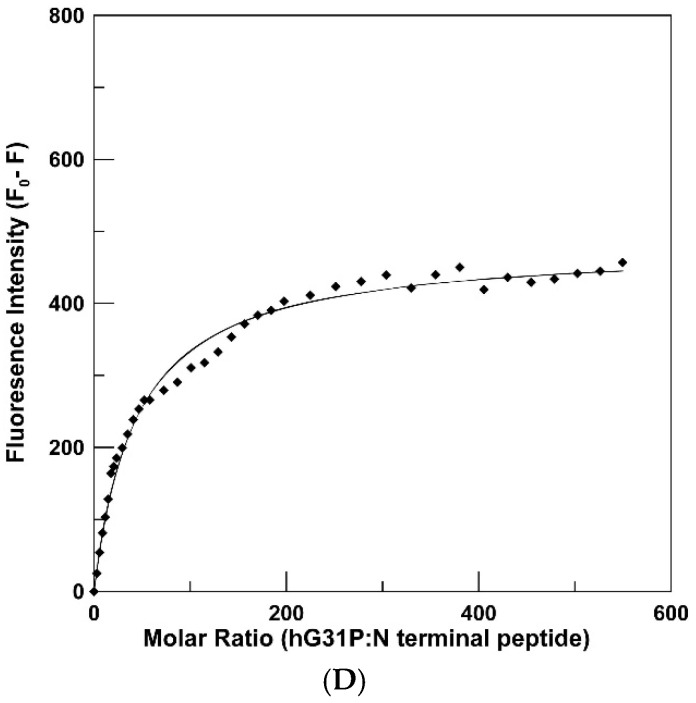
(**A**) Overlay of the ^1^H-^15^N HSQC spectrum of hG31P (red) and the hG31P/CXCR1 N-terminal peptide complex (black). Cross-peaks with significant shifts are labeled according to the residue types and numbers; (**B**) Chemical shift change versus residue number of hG31P upon CXCR1 N-terminal peptide binding. The chemical shift changes are calculated according to the equation: Δδ = [Δδ(^1^H)^2^] + [[0.2xΔδ(^15^N)]^2^]^1/2^; (**C**) Surface rendering of hG31P bound to CXCR1 N-terminal peptide. The blue shading corresponds to the residues that give rise to a change in Δδ chemical shift great than 0.04 ppm. The peptide is shown as a stick model in red; (**D**) Equilibrium binding of hG31P to the N-terminal peptide. Solid squares are the hG31P dependent fluorescence signal intensity at 516 nm (*K_d_* = 48 ± 5 μM). Solid line represents curve fitting of the single binding site model.

CXCR1 and CXCR2 are the specific receptors of CXCL8. N-terminal peptides of CXCR1 have been shown to prevent CXCL8 from binding to CXCR1 and hence act as inhibitors of the signaling cascade [18,19]. In this study, the N-terminal peptide derived from CXCR1 [10] was titrated into ^15^N-labeled hG31P to a final molar ratio of 3:1 (Figure 5A). Residues Leu15, Asn16, Phe17, Thr18 and Gly19 of the wild-type CXCR1 N-terminal sequence were replaced by a single 6-aminohexanoic acid moiety in this N-terminal CXCR1 peptide [19]. The reason to choose this peptide was due to its small size and potency of inhibition of CXCL8 receptor binding [19]. It also allows us to compare the NMR results with the previous peptide/CXCL8 complex studies [10]. Comparison of the ^1^H-^15^N cross-peaks in the free and the peptide bound forms of hG31P indicates that complex formation causes chemical shift changes for a discrete set of resonances (Figure 5B). Figure 5C was generated based on the averaged structure of hG31P and the residues involved in peptide induced chemical shifts. The orientation of the N-terminal peptide was placed onto hG31P structure based on the structural model of the peptide/CXCL8 complex [10].

The dissociation constant (K_d_) of the N-terminal peptide to hG31P was determined to be 48 μM by fluorescence spectroscopy (Figure 5D). Similar dissociation constant was reported in the peptide/CXCL8 complex structural studies [10]. It needs to mention that, although NMR chemical shifts indicate possible binding sites between hG31P and the N-terminal peptide, structural details of the peptide/hG31P complex can only be obtained by other NMR techniques such as intermolecular NOEs [10] and STD NMR techniques [20].

### 2.4. Biological Importance

**Figure 6:**
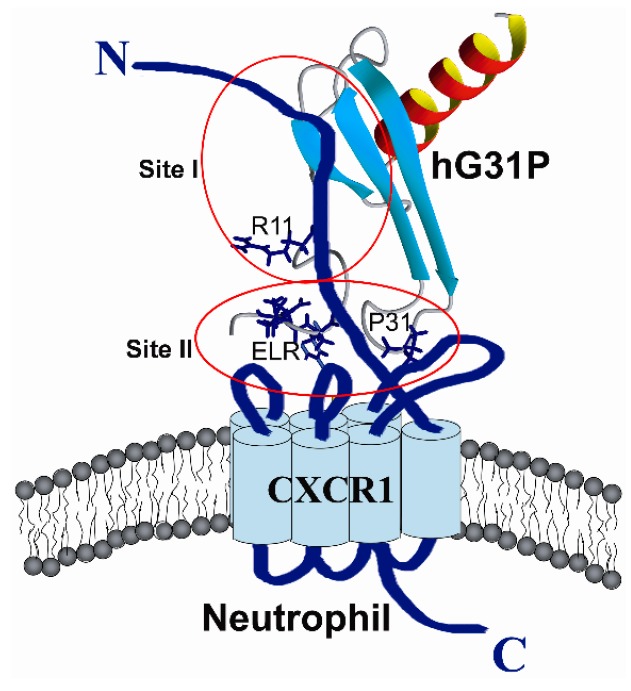
Model for the binding of hG31P (ribbon structure) to the CXCR1 receptor (blue) on a neutrophil. The side chains of the N-terminal ELR, R11, P31 residues of hG31P are shown in sticks (blue). Site I indicates the interactions between the hG31P N-loop residues and receptor N-domain, and site II indicates the hG31P N-terminal and 30 s loop residues and receptor exoloop residues. The displacement of the N-terminal ELR region and the 30 s loop and the change of the 30 s loop conformation may affect its binding and activation to the CXCR1 receptor thus inhibit the neutrophil chemotaxis signaling pathway.

Structure-function studies using site-specific mutagenesis and generation of chimeric chemokines by swapping identical domains have indicated that, in CXCL8, the N-terminal and 30 s loop residues are important for receptor binding affinity and activation, and the N-loop residues are essential for receptor binding affinity and selectivity [6,7,8,9,10]. A two site mechanism of chemokine-receptor interaction has been proposed recently [21]. It has been proposed that binding involves interactions between the ligand N-loop residues and receptor N-domain (site-I), and ligand N-terminal and 30 s loop residues and receptor exoloop residues (site-II) (Figure 6).

In this paper, we have found that, in hG31P, the K11R and G31P mutations of CXCL8 force H33 to point out from the 30 s loop and hence affect its binding and activation of the CXCR1 receptor (Figure 4A). We have also compared the sequences and structures of CXCL1 to CXCL10 (Figure 2A and Figure 4C). Surprisingly, according to the determined structures and functions, molecules with residue 33 pointing toward the 30 s loop all possess CXCR1 and/or CXCR2 activity. On the other hand, molecules with residue 33 pointing out from the 30 s loop have no CXCR1/CXCR2 activity. For example, CXCL4 (PF-4) has the same G31–P32–H33 loop sequence and similar loop structure (Figure 4B) comparing with CXCL8 but does not have the N-terminal ELR motif. The CXCR1 binding and activation activity of CXCL4 was gained while the N-terminal ELR motif from CXCL8 was grafted [22].

**Figure 7:**
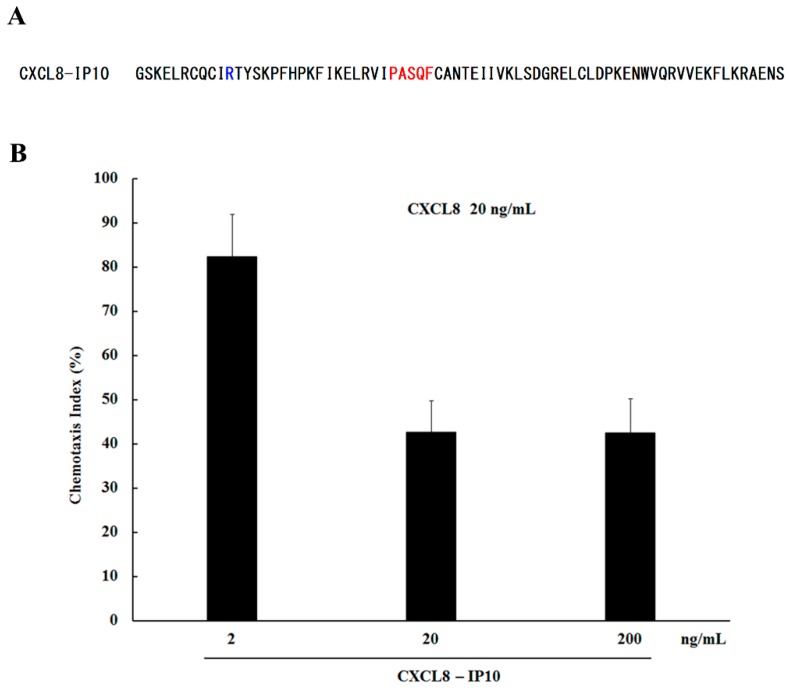
(**A**) Amino acid sequences of the designed CXCL8-IP10 molecule. Residues derived from CXCL8 are shown in black, residues derived from CXCL10 (IP10) are shown in red, K11R mutation site is shown in blue; (**B**) CXCL8-IP10 effectively antagonizes human neutrophil responses to ELR-CXC chemokine CXCL8.

As for CXCL10 (IP10), the 30 s loop sequences and structure (Figure 4C) as well as the N-terminal motif of CXCL10 are different from CXCL8. Previous studies have shown that a hybrid molecule of CXCL10 which both its N-terminal motif and the 30 s loop were replaced by sequences of CXCL8 can bind to CXCR1 and activate its activity [9]. Furthermore, K11R mutation can increase the binding affinity of hG31P to CXCR1/CXCR2 and hence compensate the potential loses of binding affinity to the receptors caused by the G31P mutation [9], The above-mentioned structural differences and the ELR motif modification results make us envision that a hybrid molecule CXCL8-IP10 with the structural frame of CXCL8 and the 30 s loop of CXCL10 may still have the required receptor binding abilities but not the neutrophil attraction properties (Figure 7A).

Indeed, for the expressed and purified CXCL8-IP10, we have tested its abilities to antagonize activation of chemotactic responses of purified human neutrophils by ELR-CXC chemokines [23]. The results indicated that CXCL8-IP10 successfully inhibited human neutrophil chemotactic responses induced by CXCL8 (Figure 7B).

In summary, we have determined the solution structure and the CXCR1 N-terminal peptide binding sites of hG31P. We have found that the displacement of the 30 s loop and the N-terminal region and change of the loop conformation (especially H33) of hG31P may affect its binding to the CXCR1 receptor and inhibit human neutrophil chemotactic responses induced by ELR-CXC chemokines. To further understand the mechanism of such structural changes, a mutated molecule CXCL8-IP10 was designed, expressed, and purified based on the structural studies of hG31P. It was demonstrated that CXCL8-IP10 successfully inhibited human neutrophil chemotactic responses induced by CXCL8 and the studies of more biological functions of this designed molecule are currently undergoing in our laboratory.

## 3. Materials and Methods

### 3.1. Protein Preparation and Purification [24]

The pET-22b plasmid with the CXCL8_(3–72)_K11R/G31P (hG31P) sequence incorporated was transformed into *E. coli* (strain BL21(DE3)). These transformed cells were inoculated onto a LB agar plate containing ampicillin at 37 °C for 12 hours, and then the colonies were selected and incubated. The results of the incorporation of hG31P sequence into the pET-22b plasmid were checked by DNA sequencing. The uniform ^15^N-labeled and/or ^15^N/^13^C-labeled samples were expressed in cells grown in M9 minimal media containing ^15^NH_4_Cl and/or (U-^13^C) glucose. Cell pastes derived from 1 liter of culture were suspended in Tris buffer (100 mL) with high salt concentration (50 mM Tris, 1 mM EDTA, 700 mM NaCl, 1 mM PMSF, pH 8.0). Lysozyme (200 μg/mL) and TritonX-100 (0.5%) were then added. A high-pressure homogenizer (EmulsiFlex C3, AVESTIN, Ottawa, ON, Canada) was used to lyse the suspended cells. The cell lysates were bathed in 80 °C water for 10 min, and immediately cooled in 0 °C ice water for 30 min. The result was checked by SDS PAGE. The supernatant was collected after centrifuged at 14,000× *g* for 15 min at 4 °C and followed by dialysis using a cellulose tubular membrane (Cellu·Sep T1 (Uptima), Membrane Filtration Products, Seguin, TX, USA) against 20 mM citrate buffer (pH 6.0) at 4 °C for 8 hours. The supernatant and citrate buffer ratio was 1:10. The residual pellets were dissolved by 100 mL 8 M urea with 1% TritonX-100 for SDS PAGE analysis. The dialyzed supernatant was loaded onto a SP Sepharose fast flow column and washed using the citrate buffer (pH 6.0) with different salt gradients. Finally, the hG31P protein was eluted with 20 mM citrate and 600 mM NaCl buffer. An Amicon Stirred Cell with a YM1 membrane was used to concentrate the eluted hG31P protein. The purity of the protein was confirmed by HPLC and mass spectroscopy. The final protein concentration for NMR study was 2 mM in 90%H_2_O/10%D_2_O.

### 3.2. Neutrophil Chemotaxis Assay

Neutrophil chemotaxis was assessed using modified Boyden chamber microchemotaxis assays. Briefly, leukocytes obtained from human peripheral blood were fractioned on standard density gradients, and the neutrophils harvested from the bottom of the gradients and cleared of contaminating red blood cells by hypotonic lysis. The purified neutrophils were suspended at 2 × 10^6^/mL in PBS^+^ (phosphate-buffered saline [PBS; pH 7.4], 1.2 mM MgCl_2,_ 5 mM KCl, 0.5 mM CaCl_2_, 5 mM glucose, and 0.1% bovine serum albumin). The chemoattractants (e.g. CXCL8), either alone or together with CXCL8-IP10, were placed in the bottom compartment of the Boyden chamber wells and purified neutrophils in the upper compartment, with the two compartments separated by polyvinylpyrrolidone-free, 5 µm pore-size polycarbonate filters. In preliminary experiments we confirmed that CXCL1, CXCL5, and CXCL8 induced maximal neutrophil chemotactic responses at concentrations of 100, 100, and 10 ng/mL, respectively. After incubation for 20 minutes at 37 °C in a 5% CO_2_ atmosphere, the cells that had migrated into the filters were fixed and stained using a Diff-Quick kit. The numbers of cells responding in each well were enumerated by direct counting of at least five 40× objective fields, and the results expressed as the mean number of cells per 40× field ± SEM.

### 3.3. NMR Spectroscopy

All NMR data used in the structural analysis were acquired with Avance 600 or 500 MHz spectrometers (Bruker, Silberstreifen, Germany) equipped with triple-resonance probes at 25 °C. ^1^H-NMR data were referenced to the ^1^H resonance frequency of DSS; ^13^C and ^15^N resonances were referenced indirectly by multiplying the proton frequency by 0.25144953 for ^13^C and 0.101329118 for ^15^N [25,26]. The NMR experiments performed included 2D ^1^H-^15^N HSQC, ^1^H-^13^C HSQC, 3D ^15^N-NOESY-HSQC, HNCO, HN(CA)CO, HN(CO)CA, HNCA, CBCA(CO)NH, and HNCACB for backbone assignments, and ^15^N-TOCSY-HSQC, HCC(CO)NH-TOCSY, HCCH-TOCSY, HCCH-COSY, HBHA(CO)NH for side chain assignments [27]. All spectra were processed with the program XWIN-NMR 2.6 (Bruker, Silberstreifen, Germany) and NMRPipe and analyzed using NMRView 5.04 (Molecular Systems, Rahway, NJ, USA).

### 3.4. Structure Calculation

NOE based distance restraints were collected from analysis of 3D ^15^N-edited NOESY-HSQC and ^13^C-edited NOESY-HSQC spectra recorded with mixing time of 150 and 80 ms respectively. The φ and ψ angles were obtained based on ^3^*J*_HNHα_ coupling constants derived from HNHA experiment and predicted from TALOS [28]. Hydrogen bond restraints were included in calculations only if the amide protons were slowly exchanging and if the β-strand inter-strand NOE cross-peaks were observed. The structure calculations were carried out using X-PLOR 3.851 [29] program on a SuSE Linux 7.3 PC. The best 20 lowest energy structures were further analyzed with MOLMOL [30] and PROCHECK-NMR [31]. The coordinates of both the representative structure and the family of structures have been deposited at the Brookhaven Protein Data Bank (access number 2RPY).

## Figures and Tables

**Table 1:** Summary of structural constrains and structure statistics.

NOE Restraints	2343
Intraresidue (|i-j|=0)	532
Sequential (|i-j|=1)	669
Medium range (2≦|i-j|≦4)	352
Long range (|i-j|>4)	465
Hydrogen bond constraints	120
Dihedral angles ^a^	106
Intermolecular	99
Final energy (kcal mol^−1^)	
E (total)	209.8468 ± 13.8168
E (bond)	10.20659 ± 1.57751
E (angle)	100.5601 ± 5.99895
E (improper)	9.874589 ± 0.97132
E (van der Waals)	51.37347 ± 5.53424
E (NOE)	37.72119 ± 3.85691
E (cdih)	0.110884 ± 0.24450
Structural Statistics (20 structures)	
NOE violations, number > 0.3 Å	0
Dihedral angel violations, number > 5°	0
RMSD for geometrical analysis	
Bond lengths (Å)	0.002 ± 0.0002
Bond angles (degree)	0.383 ± 0.0116
Impropers (degree)	0.228 ± 0.0144
Atomic RMSD for protein ^b^	
All heavy atoms	1.38 ± 0.08
Backbone	0.68 ± 0.09
Ramachandran statistics ^b^	
Most favoured region (%)	88.8
Additionally allowed (%)	11.1
Generously allowed (%)	0.1
Disallowed (%)	0.0

^a^ Dihedral angles were predicted from the program TALOS. ^b^ For residues 7–31 and 37–72 in the dimer structure.
